# Bipolar resistive switching in p-type Co_3_O_4_ nanosheets prepared by electrochemical deposition

**DOI:** 10.1186/1556-276X-8-36

**Published:** 2013-01-19

**Authors:** Adnan Younis, Dewei Chu, Xi Lin, Jiunn Lee, Sean Li

**Affiliations:** 1 School of Materials Science and Engineering, University of New South Wales, Sydney, New South Wales, 2052, Australia

**Keywords:** Electrochemical deposition, Resistive switching, Nanosheets

## Abstract

Metal oxide nanosheets have potential applications in novel nanoelectronics as nanocrystal building blocks. In this work, the devices with a structure of Au/p-type Co_3_O_4_ nanosheets/indium tin oxide/glass having bipolar resistive switching characteristics were successfully fabricated. The experimental results demonstrate that the device have stable high/low resistance ratio that is greater than 25, endurance performance more than 200 cycles, and data retention more than 10,000 s. Such a superior performance of the as-fabricated device could be explained by the bulk film and Co_3_O_4_/indium tin oxide glass substrate interface effect.

## Background

Resistive random access memory (RRAM) is one of the emerging non-volatile memory technologies. It is composed of a thin insulator layer sandwiched between two metals (MIM) that have competitive advantages of greater writing and reading speed, smaller size, and low programming voltage over phase-change RAM [1], magnetoresistive RAM [2], flash memory [3], and ferroelectric RAM [4]. Resistive switching (RS) with different switching behaviors, including bipolar, unipolar, and threshold switching, have been reported in various n-type metal oxides (e.g., perovskite oxides [5,6] and transition metal oxides [7-11]). As to the resistive switching mechanism, compared with n-type oxides where oxygen vacancies play a crucial role in the switching process, understanding the resistive switching conduction nature of p-type oxides such as cobalt oxides and nickel oxides, which exhibit excellent memory characteristics [12], is rather scarce. This is due to the lack of direct experimental evidence to verify the resistive switching conduction characteristics.

Two-dimensional nanosheets are considered to be excellent candidates for future nanoelectronic applications [13,14]. Such nanostructures and their electronic states play an important role in realizing the innovative electronic, optical, and magnetic functionalities. For example, the operation of almost all semiconducting devices relies on the application of two-dimensional interfaces. To date, various nanosheets have attracted increasingly fundamental research interest because of their potential to be used for different applications like electrochemical capacitors [15,16] and super capacitors [17-19]. However, the resistive switching properties in p-type oxide nanosheets have remained much less explored.

In this work, we developed a facile approach to fabricate high-quality p-type Co_3_O_4_ nanosheets with excellent resistive switching properties. Morphology-controlled Ag nanostructures were also synthesized electrochemically by Liang et al. [20]. The bulk film and cobalt oxide/indium tin oxide (ITO) interface effect was studied in detail. Furthermore, the effect of Au top electrode was investigated to verify the origin of resistive switching properties in these devices.

## Methods

Co_3_O_4_ nanosheets were prepared by electrochemical deposition, using an Autolab 302N electrochemical workstation (Metrohm, Utrecht, The Netherlands). A standard three-electrode setup in an undivided cell was used. ITO (9.7 Ω, 1.1 × 26 × 30 mm; Asahi Glass Corporation, Tokyo, Japan) was used as the working electrode, while platinum foil (0.2 × 10 × 20 mm) was used as the counter electrode. The distance between the two electrodes was 30 mm. The reference electrode was an Ag/AgCl electrode in 4 M KCl solution, against which all the potentials reported herein were measured.

The ITO substrates were first cleaned by detergent, then rinsed well with ethanol and DI water and then electrodeposited in a solution of 0.1 M Co(NO_3_)_2_.6H_2_O at −0.8 V for 20 min at 70°C. The as-deposited films were post-annealed in air at 300°C for 1 h with heating and cooling rates of 5°C/min. The phase composition of the samples was determined by X-ray powder diffraction (PANalytical Empyrean (Almelo, The Netherlands with CuKα). The morphologies and microstructure of the samples were characterized by scanning electron microscopy (Nova NanoSEM 230, FEI, Hillsboro, OR, USA)and transmission electron microscopy (TEM; Philips CM200, Amsterdam, Netherlands), respectively. To measure the electrical properties of the films, Au top electrodes were patterned and deposited by sputtering using a metal shadow mask. Voltage–current curves of the films were measured using an Autolab 302 N electrochemical workstation controlled with Nova software (with a possible error in current and voltage values as ±5%). All measurements were repeated at least twice to confirm the results. In the measurement, the working electrode and sensor electrode were connected to the top Au electrode, and the reference and counter electrodes were connected to the ITO substrate.

X-ray photoelectron spectroscopy (XPS) was performed with an ESCALAB250Xi spectrometer using a monochromatized Al K alpha X-ray source (*h*V) 1,486.6 eV with 20 eV pass energy. Hall effect measurements were carried out by the Accent HL5500PC (Nanometrics, Milpitas, CA, USA). All measurements were performed at room temperature.

## Results and discussion

**Figure 1:**
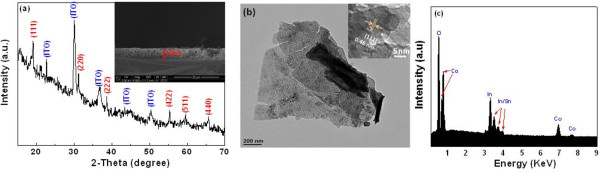
**Co_3_O_4_ nanosheets deposited on the ITO substrate.** (**a**) X-Ray diffraction pattern (inset, cross-sectional image). (**b**) TEM image of the mesoporous sheets (inset, HRTEM with lattice spacing). (**c**) Energy-dispersive X-ray spectroscopy (inset, surface morphology).

Figure 1a shows the XRD pattern of Co_3_O_4_ nanosheets deposited on the ITO substrate. All peaks are assigned to the cubic lattice of Co_3_O_4_. The diffraction data are in a good agreement with JCPDS file no. 9–418 with no CoO or other impurities detected. The cross-sectional SEM image of the sample was shown in the inset of Figure 1a, where the nanosheet with a thickness of approximately 234 nm can be clearly seen.

The detailed microstructures of the Co_3_O_4_ nanosheets were characterized with TEM. Figure 1b represents typical TEM images of Co_3_O_4_ nanosheets. The HRTEM image shown in the inset of Figure 1b clearly demonstrates lattice fringes with a d-spacing of 0.46 nm (111), matching well with the XRD pattern. To further elucidate the composition, energy-dispersive X-ray spectroscopy was used to determine the nominal stoichiometric atomic ratio of Co and O, as shown in Figure 1c.

**Figure 2:**
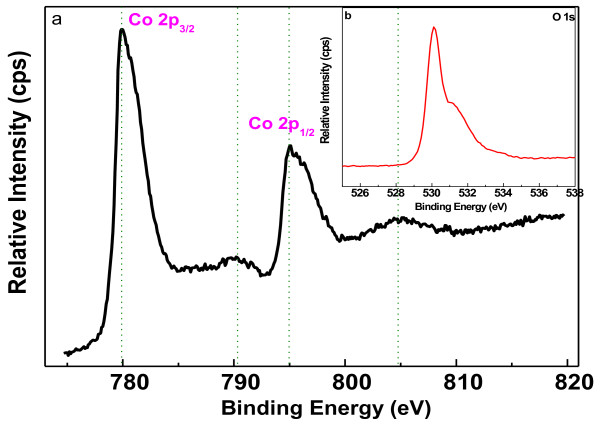
Co 2*p* (a) and O 1*s* (b) XPS spectra of Co_3_O_4_ sample.

The chemical composition of the film was investigated by XPS analysis. The spectra (Co 2*p* and O 1*s*, as shown in Figure 2) were acquired and processed using standard XPS peak fitting. Two peaks at binding energies of 780 and 795.1 eV were observed from the Co 2*p* spectra. The tetrahedral Co^2+^ and octahedral Co^3+^ contributed to the spin-orbit doublet 2*p* spectral profile of Co_3_O_4_[21]. The relatively sharp peak widths correspond to 2*p*_1/2_ to 2*p*_3/2_ with separation of 15.1 eV, and the weak satellite structure found in the high binding energy side of 2*p*_3/2_ and 2*p*_l/2_ transitions indicate the co-existence of Co(II) and Co(III) on the surface of the material. The Co 2*p* spectrum is well consistent with the XPS spectrum of Co_3_O_4_[22-24].

The O 1*s* spectra of the sample was also presented in the inset of the same figure The peak at around 530 eV is due to lattice O, while the peak at about 531.6 eV can be attributed to the low coordinated oxygen ions (chemisorbed oxygen) at the surface [25].

**Figure 3:**
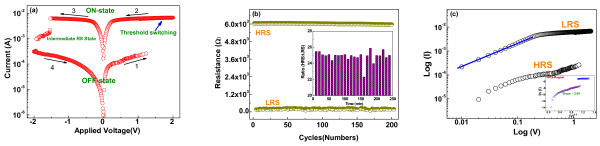
**RS properties of the Au/Co_3_O_4_/ITO memory cells.** (**a**) Typical bipolar resistance switching *I*-*V* curves of the Au/Co_3_O_4_/ITO cells. (**b**) Electrical pulse-induced resistance switching of the Au/Co_3_O_4_/ITO memory cell at room temperature for 60 s, (inset, data retention of Au/Co_3_O_4_/ITO memory cell for >10^4^ s), and (**c**) *I*-*V* curves on log scale.

Figure 3a presents the typical current–voltage (*I*-*V*) characteristics of RRAM cell with the Au/Co_3_O_4_/ITO structure, measured by sweeping voltage, at a speed of 1 V/s, in the sequence of 0 → 2 → 0 → −2 → 0 V. During the measurements, the bias voltages were applied to the gold top electrode with ITO bottom electrode as ground. By steady increase of the positive voltages imposed on the RRAM cell, a pronounced change of resistance from the high-resistance state (HRS/OFF) to the low-resistance state (LRS/ON) was observed at about 1.05 V, which is called as the SET’ process, and then the device was set in threshold switching mode (no change in current after this voltage).

Subsequently, an opposite ‘RESET’ process could also be cited, with the voltage sweep to negative values bringing the device first to an intermediate switching state at −1.53 V that increased up to −1.93 V and, after that, completely to OFF state. The sample exhibits a typical bipolar nature of resistive switching. It shows that the positive biasing can be utilized for writing data, while the negative biasing for erasing.

The electrical stabilities of the Au/Co_3_O_4_/ITO memory device at LRS and HRS have been examined using endurance and retention test. It was observed that the stable HRS and LRS states were maintained with an *R*_OFF_/*R*_ON_ ratio of about 25 for 200 pulses, and almost no degradation in the resistance ratio was observed during pulse measurements, as shown in Figure 3b. The device well maintained its switching states (HRS to LRS ratio) for more than 10 s [4], which indicates that Au/Co_3_O_4_/ITO memory cell can be qualified as a RRAM device due to its decent retention time.

To further investigate the origin of switching behavior, the *I*-*V* curves were replotted on a log-log scale, as shown in Figure 3c. The high conductive state (LRS) slightly follows the ohmic conduction behavior. However, the low conductive state (HRS) was found to follow an ln *I* vs. *V*^0.5^ behavior with a slope of 2.6 in the inset of Figure 3c, which leads to following a Schottky-type conduction emission.

For resistive switching operations in these devices, the distribution of oxygen ions and its motion can be discussed on the basis of an ionic model [26-28] that describes the hopping mechanism of O^2−^ ions between different potentials. In our device, ITO used as a bottom electrode can act as a source/reservoir of oxygen ions [29], and their gradient may produce some diffusion flux (from higher concentration to lower concentration). So, the diffusion coefficient (denoted as *D*) is expressed as [30]

(1) D=D0exp−Eakt

where *D*_o_ is the diffusion constant, *E*_a_ is the activation energy of oxygen vacancy/defect diffusion, *k* is Boltzmann's constant, and *T* is the absolute temperature.

**Figure 4:**
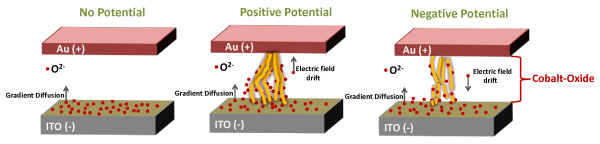
**Schematic of the Co-rich metallic filament in Co_3_O_4_.** With oxygen gradient-induced drift and the field-induced diffusion motions of the oxygen ions (bulk film effect).

Hence, the dynamics of oxygen concentration (*V*_o_) could be described by taking into account both diffusion (thermal) and drift (electric) effects. Thus, the net continuity equation with its time and displacement dependence is expressed as [30]

(2) $$\frac{\partial V_{0}}{\partial t}=\;D\frac{\partial^{2}V_{0}}{\partial x^{2}}+\;\upsilon \frac{\partial V_{0}}{\partial x}-\frac{V_{0}}{\tau }$$

where the left side of Equation 2 represents time-dependent evolution of oxygen concentration (*V*_o_), *D* is the diffusion coefficient, *υ* is the drift velocity, and *τ* represents the recombination time of oxygen ions with metallic cobalt to offset the contribution from oxygen vacancies. In the Au/Co_3_O_4_/ITO device, the applied electrical field generates the drift motion of the oxygen ions, thus inducing the local reduction of Co_3_O_4_ with the formation of metallic conducting filaments. With further increase of potential (higher voltage), a substantial Joule heating effect may be generated in the device, which promotes oxygen ion diffusion from ITO into Co_3_O_4_. As a consequence, the migration of oxygen ions may reduce oxygen vacancies and generate Co vacancies simultaneously, which weaken the conducting filaments first and then shatter (due to further joule heating) them by setting the device to threshold switching state [31,32], as illustrated in Figure 4. The migration of oxygen ions which transforms the device from switching memory to threshold state was observed for positive voltages only. For a negative applied bias, the oxygen ion diffusion process starts deceleration that results in filament breaking (intermediate switching state). At a higher negative potential, the diffusion became negligible with majority of ruptured conducting filaments, hence no observable threshold switching state. This polarity dependence implies that the switching transition hinges on the delicately balanced migration of oxygen ions, which must be carefully considered to achieve reliable device operations.

In addition to bulk film effect, the interface between ITO of the bottom electrode (n-type) nanosheet and cobalt oxide (p-type) is also critical to explain switching characteristics Consider the interface as a classical p-n junction with negatively charged electrons or oxygen ions in cobalt oxide and positively charged electrons or oxygen ions in oxygen vacancies in ITO (acting as minority charge carriers in both regions) accumulate at the interface to form a depletion layer. Under forward voltage sweep, these minority charge carriers start moving away from the junction, tending to decrease the width of depletion region with a sudden increase in current (high conduction state or LRS), as shown in Additional file 1: Figure S2. The negative applied voltage facilitates the migration of minority charge carriers in both regions towards the junction, which results in the increase of depletion layer causing decrease in current (low conduction state or HRS).

To exclude the possible metal/metal oxide (Au/Co_3_O_4_ layers) interface effect (Au used as a top electrode), a test sample without a gold top electrode was also investigated, and the results are shown in Figure S3. It is interesting to note that the RS properties of the device were quite repeatable and similar to the device with Au as the top electrode. This interesting behavior indicates that Au has no significant effects in the resistive switching properties of Co_3_O_4_ except for acting as an electrical contact of these devices.

## Conclusions

In summary, Co_3_O_4_ thin films with nanosheet structure were prepared with a facile electrochemical deposition method. Excellent bipolar resistance switching properties, stable endurance, and retention performance for more than 4 h without observable degradation were achieved. The oxygen ions/vacancies throughout the as-deposited film and interface with minority charge carrier effect are responsible for the switching behavior. Furthermore, the effect of Au top electrode was investigated to verify the origin of resistive switching properties in these devices. The present work demonstrates that these structures have the potential for next-generation non-volatile memory applications.

## Competing interests

The authors declare that they have no competing interests.

## Authors’ contributions

AY and DC carried out the sample preparation, participated on its analysis, performed all the Analyses, and wrote the paper. XL and JL helped perform the XRD and EDS analyses. SL guided the study and participated in the paper correction. All authors read and approved the final manuscript.

## Supplementary Material

Additional file 1 **Supporting information.** Contains supporting information (Figures S1, S2, and S3). Click here for file
