# Supplementary material for: Bipolar resistive switching in p-type Co3O4 nanosheets prepared by electrochemical deposition
Source: Nanoscale Res Lett. 2013 Jan 19;8(1):36. doi: 10.1186/1556-276X-8-36 (PMC3564723; doi:10.1186/1556-276X-8-36)
Supplement: Additional file 1 — Supporting information. Contains supporting information (Figures S1, S2, and S3). [file 1556-276X-8-36-S1.docx]

**Bipolar Resistive Switching in *p*-type Co_3_O_4_ Nanosheets Prepared by Electrochemical Deposition**

**Adnan Younis, Dewei Chu^^[[1]](#footnote-1)^*^ Xi Lin, Jiunn Jieh Lee and Sean Li**

*School of Materials Science and Engineering, University of New South Wales, Sydney, 2052, NSW, Australia*

***Supporting Information***

Figure S1 Schematic of conduction mechanism in Co_3_O_4_/ITO p-n junction (interface effect)

Figure S2: (a) Bipolar resistance switching *I*–*V* curves of the Co_3_O_4_/ITO device. ((inset data retention Au/Co_3_O_4_/ITO memory cell for (>10^4^s)), (c) I-V curves on log scale

Figure S3: (b) Electrical pulse-induced resistance switching of the Co_3_O_4_/ITO memory cell at room temperature for 60 sec,

The conductivity nature of Co_3_O_4_ film was confirmed by observing hall co-efficient, carrier mobility and sheet carrier concentration as (+0.0269 m^2^/C), (18.3 cm^2^/v-sec) and 2.319x10^16^ cm^-2^ respectively.

1. *Corresponding Author, Tel.: +61 (0)2 9385 9934; Fax: +61 (0)2 9385 6565

   E-mail address: d.chu@unsw.edu.au [↑](#footnote-ref-1)
